# Secretory protein beta‐lactoglobulin in cattle stable dust may contribute to the allergy‐protective farm effect

**DOI:** 10.1002/clt2.12125

**Published:** 2022-02-12

**Authors:** Isabella Pali‐Schöll, Rodolfo Bianchini, Sheriene Moussa Afify, Gerlinde Hofstetter, Simona Winkler, Stella Ahlers, Theresa Altemeier, Hanna Mayerhofer, Karin Hufnagl, Anna D. J. Korath, Christina Pranger, Raimund Widhalm, Stephan Hann, Thomas Wittek, Anne Kasper‐Giebl, Luis F. Pacios, Franziska Roth‐Walter, Donata Vercelli, Erika von Mutius, Erika Jensen‐Jarolim

**Affiliations:** ^1^ The Interuniversity Messerli Research Institute of the University of Veterinary Medicine Vienna, Medical University Vienna and University Vienna Vienna Austria; ^2^ Institute of Pathophysiology and Allergy Research; Center of Physiology, Pathophysiology and Immunology; Medical University Vienna Vienna Austria; ^3^ Laboratory Medicine and Immunology Department Faculty of Medicine Menoufia University Menoufia Egypt; ^4^ Institute of Medical Genetics Medical University of Vienna Vienna Austria; ^5^ Karl‐Landsteiner Private University for Health Sciences Krems Austria; ^6^ Division of Analytical Chemistry, Department of Chemistry, University of Natural Resources and Life Sciences, BOKU‐Vienna Vienna Austria; ^7^ University Clinic for Ruminants University of Veterinary Medicine Vienna Vienna Austria; ^8^ Institute of Chemical Technologies and Analytics, TU‐Wien Vienna Austria; ^9^ Centro de Biotecnología y Genómica de Plantas (CBGP, UPM‐INIA), Campus de Montegancedo UPM and Departmento de Biotecnología‐Biología Vegetal, ETSIAAB, Universidad Politécnica de Madrid (UPM) Madrid Spain; ^10^ Arizona Respiratory Center University of Arizona College of Medicine Tucson Arizona USA; ^11^ Asthma and Allergy Department Dr. von Hauner Children's Hospital University of Munich Munich Germany

**Keywords:** BLG, cattle, farm effect, immunomodulation, stable, zinc

## Abstract

**Background:**

Growing up on a cattle farm and consuming raw cow's milk protects against asthma and allergies. We expect a cattle‐specific protein as active component in this farm effect.

**Methods:**

Dust was collected from cattle and poultry stables and from mattresses of households. Urine was obtained from cattle, and ambient aerosols were sampled. Samples were analysed for BLG by SDS PAGE/immunoblot and mass spectrometry, and for association with metals by SEC‐ICP‐MS. PBMC of healthy donors were incubated with BLG +/− zinc, and proliferation and cytokines determined. BALB/c mice were pre‐treated intranasally with stable dust extract containing BLG or depleted of BLG, and subsequent allergy response after sensitization was evaluated on antibody and symptom level.

**Results:**

A major protein in dust from cattle farms and ambient air was identified as BLG. Urine from female and male cattle is a major source of BLG. In dust samples, BLG was associated with zinc. *In vitro*, zinc‐BLG provoked significantly lower proliferation of CD4^+^ and CD8^+^ cells while inducing significantly higher levels of IFN‐γ and IL‐6 than the apo‐BLG devoid of zinc. *In vivo*, pre‐treatment of mice with dust extract containing BLG resulted in lower allergy symptom scores to BLG and unrelated Bet v 1 than pre‐treatment with extract depleted of BLG. These *in vitro* and *in vivo* effects were independent of endotoxin.

**Conclusion:**

The lipocalin BLG is found in large amounts in cattle urine, accumulates in bovine dust samples and is aerosolized around farms. Its association with zinc favorably shapes the human cellular immune response towards Th1‐cytokines *in vitro*. BLG together with zinc in stable dust protects mice from allergic sensitization. BLG with its associated ligands may in an innate manner contribute to the allergy‐protective farm effect.

## 1 BACKGROUND

Living on a farm especially with traditional farming conditions results in a higher encounter of factors stimulating the innate immune system, for example endotoxin^1^ or N‐glycolylneuraminic acid (Neu5Gc).^2^ These factors can also be found in indoor dust samples of farm‐associated homes. The protective effect of these indoor dust samples was seen in lower prevalence numbers of children affected by asthma and allergies,^3^ and was further proven in a murine animal model of OVA‐induced asthma,^1^ where the ubiquitin‐modifying enzyme A20 was described as a responsible factor.^4^ The connection between farm life and asthma‐protection was also shown by differences in the indoor microbiota composition between farm and non‐farm homes of Finnish birth cohorts.^5^

However, not all farms were equally protective against asthma or allergies: especially cattle and pig farming provide a beneficial environment,^6^, ^7^ in addition to consumption of raw cow's milk being a protective factor for asthma.^8^ The syllogism for us was that also species‐specific animal‐derived proteins might play a role in the protective farm effect. Our work is thus complementary to other studies focusing on farm‐protective effects associated with stable microbiota.

We hypothesized that the bovine beta‐lactoglobulin (BLG) might be an interesting protein candidate as (i) it is secreted via milk and could likely be contained in other bovine secretions; (ii) it is a very stable protein due to numerous disulfide bridges; (iii) it is a member of the lipocalin family for which we previously could show that its immunomodulatory functions depend on the association with binding partners: while the emptied BLG (apo‐BLG) induces Th2‐responses, holo‐BLG loaded with compounds, for example iron‐quercetin or retinoic acid, results in immune resilience and prevention of allergic reactions, a result not only shown *in vitro* but also in *in vivo* animal models.^9^, ^10^, ^11^, ^12^, ^13^

To prove the relevance of these observations in real life, we specifically investigated the occurrence of BLG indoor and outdoor of cattle farms, its natural binding partners and its immunomodulatory capacity as a potential active factor in the allergy‐protective farm effect.

## 2 METHODS

### 2.1 Collection and extraction of dust samples from stables, bedrooms and environmental air around stables

Dust samples from different cattle farms were collected by different methods (Table S1, set 1) to establish collection and extraction method.

A defined set of stable dust samples (Table S1, set 2) from cattle (*n* = 14) and poultry farms (*n* = 8) was then collected by wiping stable surfaces. The respective household dust from beds of these farm inhabitants, as well as from urban households (*n* = 10) was collected by vacuum cleaner.

Ambient aerosols (total suspended particles) were collected to determine the appropriate sampling material (cellulose, teflon or quartz filters; Table S1, set 3).

For distance measurements of ambient aerosols (Table S1, set 4), cellulose filters were used; sampling time was 5‐6 days, at 0 m, 1 m, 156 m and 290 m distance from the stable. As control, unloaded cellulose filters were included in the whole extraction process and readout experiments. Control samples were collected at a high alpine site (Sonnblick Observatory, 3106 m above sea level) on quartz fiber filters. For details of dust and ambient air collection, see supplement “Collection and extraction of dust samples”.

All stable and bed dust samples were sieved through a household sieve and 50 mg of sieved dust was extracted by rotating o.n. at 4°C with PBST (pH 7.4; 2 ml for stable dust samples, 1 ml for bed dust samples). After centrifugation at 800 RCF at 4°C for 10 min, supernatants were stored at −20°C until further analysis. Aerosol samples were eluted with 3 ml PBST (40 s ultrasonic bath for quartz fiber filters), followed by rotation o.n. 4°C, 2 h RT. Supernatants were taken after centrifugation at 800 RCF at 4°C for 10 min, frozen at −20°C o.n. and lyophilized for 24 h, thereafter resolved in 300 µl deionized water and stored at −20°C until further analysis.

### 2.2 Collection of urine samples

Urine samples (Table S2) from female and male cattle (*n* = 57) were collected from different stables (corresponding to stable dust samples; Table S1, set 1) noting sex, age, lactation, feeding status and season of collection time point. Additional samples were provided by the Austrian Food Safety Agency (AGES, *n* = 34) including information about sex, age, lactation and feeding status of the animal. As control, canine (*n* = 1) and human (*n* = 1) urine samples were used. Urine samples were frozen immediately after collection.

### 2.3 BLG‐detection in stable dust, ambient aerosols and urine samples

Dust extracts as well as urine samples (undiluted) were tested for BLG content in ELISA (Bovine Beta‐Lactoglobulin Elisa Quantitation Set, Bethyl Laboratories Inc., Cat. No. E10‐125) according to manufacturer's instructions. For ambient aerosol samples, the BLG amount determined by ELISA per sampled volume (m^3^) was extrapolated to the lung volume inhaled in 24 h (0.833 m^3^/h). SDS‐PAGE was applied for subsequent staining by RotiBlue® (Carl Roth, A152) and/or silver staining, as well as BLG‐specific immunoblot (details in supplement “BLG‐detection”). For mass spectrometry (details in supplement “Mass spectometry”), bands of interest were excised manually from Roti‐blue stained 1D gels, protein identification was performed with Micro‐LC ESI MS/MS, and data bank search was performed with ProteinPilot (ParagonAlgorithm).

### 2.4 Endotoxin detection in stable dust

Endotoxin was detected in dust samples of cattle and poultry farms, extracted according to Mårtensson et al.,^14^ diluted 1:10,000 and investigated by recombinant factor C‐based test (EndoZyme® II; REF 890030, Fa. Hyglos GmbH, Bernried am Starnberger See, Germany) according to manufacturer's instructions (details in supplement “Endotoxin”).

Endotoxin levels in dust samples from different distances as well as aliquots of SDE+ and SDE‐ were measured by LAL (Limulus Amoebocyte Lysate) assay (Kinetic‐QCLTM Kinetic Chromogenic LAL Assay, 50–650U, LONZA, Switzerland) in the ISO17025‐accredited testing laboratory at the unit Water Hygiene, Institute for Hygiene and Applied Immunology, Medical University of Vienna, Austria.

### 2.5 Investigation of BLG‐association with trace elements

One cattle stable dust extract and 2 bedroom‐dust samples were randomly selected and investigated for presence of BLG, and association of binding partners (e.g. trace elements) to BLG. The measurements were performed via size exclusion chromatography combined with inductively coupled plasma mass spectrometry (SEC‐ICP‐MS). The SEC method was conducted under native conditions (75 mmol/L NaCl, pH 7.4) enabling selective separation of the intact metal/protein adduct. The on‐line combination with ICP‐MS (dynamic reaction cell with oxygen as reaction gas) allowed the sensitive detection of the proteins via sulfur (as SO^+^) and the elements aluminum, manganese, iron, nickel, copper and zinc.

### 2.6 Preparation and characterization of apo‐BLG and zinc‐BLG

Apo‐BLG (i.e. empty form) was prepared by dialyzing commercially available BLG (Sigma, L0130) against zinc‐chelator DTPA (Sigma, 32,319). Briefly, BLG (20 mg/ml) was dialyzed (SnakeSkin dialysis tubing, 3500MWCO, Thermo scientific 68,035) four times against 2 mM DTPA, pH 7.0, followed by four‐times dialyzation (including one overnight) against deionized water. For ANS‐assay, apo‐BLG (10 μM) was incubated o.n. at 4°C with different concentrations of zinc chloride (10 μM = BLG:zinc ratio of 1:1; and further 1:2, 1:10, 1:50, 1100) in TCN buffer (50 mM Tris (6.07 g/l), 10 mM CaCl2 (1.47 g/l), 150 mM NaCl (8.77 g/l), pH 7.5). ANS (8‐anilino‐1‐naphtalene sulfonic acid, Sigma A1028, 50 μM) was added for 30 min in the dark at RT. Fluorescence scan was performed (Tecan reader, infinite M200 pro) by excitation at 350 nm and emission at 350–750 nm.

Efficacy of zinc‐depletion in apo‐BLG (50 μM) and association of zinc in zinc‐BLG (50 μM:100 μM zinc) was additionally controlled in flame atomic absorption spectroscopy (details in supplement “Zinc measurement”).

### 2.7 Mapping of zinc cations on BLG epitopes

Crystal structures 4LZU and 4LZV of zinc‐BLG complexes in the presence of 2 and 20 mM zinc chloride,^15^ respectively, were retrieved from the Protein Data Bank (PDB).^16^ The biologically relevant dimeric complexes were used in our analysis. Protein Data Bank entry 4LZU has two zinc cations whereas 4LZV has three zinc ions. Residues involved in zinc‐binding in both structures were identified by searching neighborhoods of zinc cations from 2.0 Å up to 4.0 Å at 0.5 Å intervals. Sequences defining the different B‐cell and T‐cell epitope segments were located in the 3D structure of the 4LZV dimer in order to include the maximum number of zinc cations present in both PDB entries. Structural analyses as well as preparation and rendering of molecular graphics were performed with PyMOL 2.3.2^17^ and Chimera 1.13.^18^

### 2.8 Cell stimulation experiments and FACS

Apo‐BLG in comparison to zinc‐BLG was used for stimulation of PBMC from healthy donors (approved by the institutional ethics committee of the Medical University of Vienna and conducted in accordance with the Helsinki Declaration of 1975, EK number 2007/2016; *n* = 6 for proliferation; due to technical difficulties cytokines in 2 donors could not be analyzed, *n* = 4). For generating zinc‐BLG, apo‐BLG (50 μM) was incubated with zinc chloride (ZnCl_2_) in desired molar ratio of BLG:zinc (1:1, 1:2, and 1:3) for 30 min at RT. Before conducting cell stimulation experiments (details in supplement “Cell stimulation”), endotoxin in apo‐ and zinc‐BLG stock solutions was controlled by LAL‐test (Kinetic‐QCL Testkit, LONZA, 50–650U).

Life/death discrimination was performed by labelling cells with Zombie violet‐pacific blue (No 423113, Biolegend) and read‐out by FACS. Supernatants of stimulated PBMC were investigated for cytokines IL‐2, IL‐4, IL‐5, IL‐6, IL‐10, IL‐13, TNF‐α and IFN‐γ by multiplex system in FACS (LEGENDplex™ Human Th1/Th2 Panel 8‐plex, No 740729, Biolegend).

#### 2.8.1 Mouse model

Female BALB/c mice (5–7 weeks old) were treated intranasally on 2 consecutive days for 5 times with 14 days interval (A) with stable dust extract (SDE+, containing 200 ng BLG/ml extract; *n* = 5), (B) with SDE depleted of BLG via magnetic beads (Protein G Magnetic Beads, Pierce™, ThermoFischer) coupled with human IgG1 and IgG4 antibodies specific for bovine BLG^19^ acc. to manufacturer's instructions (SDE‐; BLG content below detection limit in ELISA; *n* = 5), or (C) with MQ water (*n* = 4; Figure S1A). The overall composition of dust extract (original and depleted of BLG) was controlled in SDS‐PAGE via silver staining, showing that after antigen‐specific removal of BLG via magnetic beads coated with BLG‐specific antibody, other major components remained included (data not shown). Thereafter, all animals were double‐sensitized twice i.p. with BLG (5 μg/mouse) + non‐related birch pollen allergen Bet v 1 (5 μg/mouse + 100 μL aluminum hydroxide) with an interval of 14 days. One day before sacrifice, animals were challenged i.p. with BLG (50 μg/mouse in PBS) and allergy symptoms scored by a blinded observer (details in supplement “Mouse model”). On the day of sacrifice animals were challenged i.p. with Bet v 1 (50 μg/mouse) and symptoms were scored again. Blood was drawn during i.n. treatment, before i.p. sensitization and on day of sacrifice; IgE, IgG1, IgG2a and IgA were measured in serum by ELISA (details in supplement “BLG‐detection”). Splenocytes were stimulated with apo‐BLG (25 mg/ml), Bet v 1 (25 mg/ml), and positive control concanavalin A (Con A; 2.5 mg/ml) or left unstimulated (medium control) for 96 h at 37°C and 5% CO_2_. The supernatants were harvested and stored at −20°C until further use for cytokine measurement. Cytokines were measured by ELISA acc. to manufacturer's instructions (Invitrogen sets: IL‐10 88,710,588, IFN‐γ 88,731,488, IL‐5 88,705,486 and IL‐6 88,706,488).

Experiments were carried out in accordance with the EU Directive 2010/63/EU for animal experiments (ethical approval BMBWF‐66.009/0108‐V/3b/2018).

### 2.9 Statistical analysis

Statistical analyses were performed with GraphPad Prism version 9.1 for Macintosh (GraphPad Software). Results of normally distributed data were compared with unpaired *t*‐test or Mann Whitney *U*‐test for non‐parametric data. For multiple comparison ordinary one‐way ANOVA following Tukey's multiple comparison post‐hoc test was applied. For cell viability and proliferation, Z‐normalization was performed to exclude donor‐to‐donor variation by standardizing each value from each donor; z‐norm=(x‐μ)/σ, where μ is the mean of the population of all the values from each different treatment in the respective donor, and σ is the standard deviation of the mean of the population of values from each different treatment in the considered donor. Z‐norm data were compared with repeated‐measures one‐way ANOVA with Geisser‐Greenhouse correction test and Tukey's multiple comparison post‐hoc test. Cytokines were compared by ordinary one‐way ANOVA test followed by Holm‐Sidak's multiple comparison post‐hoc test.

## 3 RESULTS

### 3.1 BLG was identified in stable dust samples and in ambient aerosols of cattle farms

**FIGURE 1:**
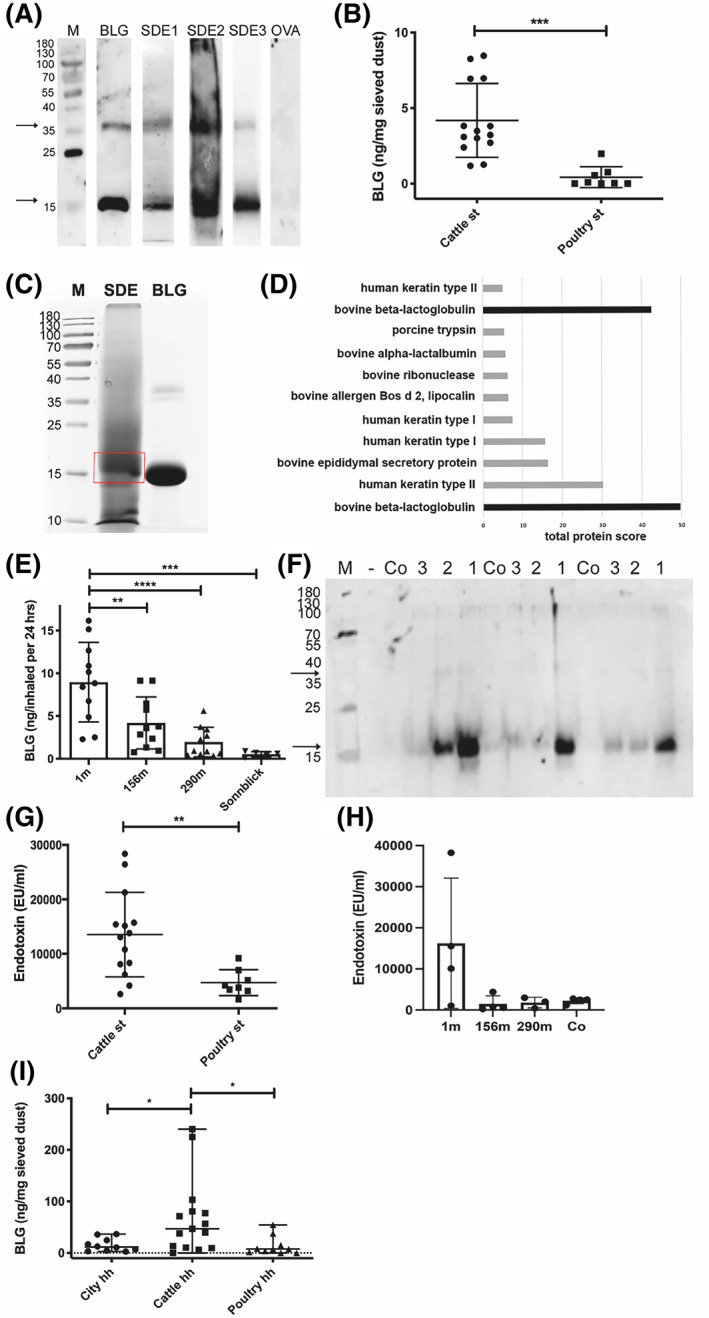
BLG and endotoxin levels in stable dust and ambient air of cattle farms. **(A)** BLG in dust of cattle stables, which was collected by different methods, detected in immunoblot: stable dust extract SDE1 (Table S1, set 1, Vet) = dust wiped from elevated surfaces; SDE2 (Table S1, Set 1, Bav) = dust deposition on cardboard box over 3 weeks; SDE3 (Table S1, set 3, Vet) = dust collected by air filtering (1 representative example of at least 3 repetitions per collection method is shown; due to time interval between examination, strips of different individual blots are shown). (**B**) Stable dust extracts (SDE), all collected by wiping, from cattle farms (*n* = 14; Table S1, set 2, C1–14) and poultry stable (*n* = 8; Table S1, set 2, P1‐8) investigated by BLG‐specific ELISA (mean +/‐ SD; representative of 3 repetitions). (**C**) BLG in stable dust (sample SDE 2) confirmed by MS/MS‐LC in SDE separated via SDS‐PAGE, stained by Roti‐Blue® and the major band around 18 kDa excised. (**D**) Protein of the excised band in MS/MS‐LC (proteins UniProtKB P02754 and B5B0D4 with difference of 2 amino acids in sequence). (**E**) BLG‐concentration in air samples at different distances from cattle stable (Table S1, set 4, *n* = 4 filter/distance), extrapolated to the human respiratory volume per 24 h, determined in ELISA (1 m = outside the stable in front of open window; 156 m and 290 m distance from cattle stable, sampled on cellulose filters; at the mountain site *Sonnblick* at 3106 m above sea level, sampled on quartz fiber filters); and (**F**) in immunoblot with bovine BLG‐specific antibodies (1 = 1 m, 2 = 156 m, 3 = 290 m, Co = empty control filter; 3 different time points from E shown). (**G**) Levels of endotoxin were determined in dust samples of cattle (*n* = 14) and poultry stable (*n* = 8) (Table S1, set 2) by LAL test, and (**H**) in dust samples collected in different distances to cattle stable. (**I**) Occurrence of BLG in different households (hh). BLG in sieved bed dust samples from beds of cattle farm households (Cattle hh. *N* = 14) , poultry farm households (Poultry hh, *n* = 8) or urban apartments (Urban hh, *n* = 10), detected by an anti‐BLG antibody in ELISA (mean of 2 repetitions). BLG (commercial beta‐lactoglobulin) = positive control; OVA (ovalbumin) and Co (empty control paper filter) = negative control. M: protein weight marker in kDa. Arrows indicate monomeric (around 18 kDa) and dimeric (38 kDa) BLG. *p < 0.05, **p < 0.01, ***p < 0.001, ****p < 0.0001

BLG could be detected as monomer (18 kDa) or dimer (36–38 kDa) in all cattle stable dust extracts (SDE), independent of method or site of collection in the stable (Figure 1A).

BLG was found at significantly higher levels in samples extracted from dust of cattle farms (*n* = 14) than in poultry stable controls (*n* = 8) via ELISA (Figure 1B).

To confirm the identity of the dominant protein in cattle stable dust, one protein extract was separated by SDS‐PAGE and the dominant band at around 18 kDa (Figure 1C) was excised and subjected to mass spectrometry. While different cattle and human proteins were found in the broader‐cut band, the dominant protein could be confirmed to be BLG (UniProtKB P02754 and B5B0D4 proteins with difference of 2 amino acids in sequence; Figure 1D).

BLG concentration in ambient aerosol samples declined with increasing distance from the cattle stable, forming a concentration gradient (1 m > 156 m > 290 m; Figure 1E,F). Samples directly collected in the stable (0 m) contained 184+/−163 ng of BLG/24‐h inhaled air volume. Control samples collected at a mountain top (*Sonnblick*) contained only marginal levels of bovine BLG (Figure 1E).

Like BLG, endotoxin levels were significantly higher in cattle stable dust than in poultry stable dust (Figure 1G). For samples collected at different distances to stable, endotoxin levels (mean ± SD) were 16,232 ± 15,841 EU/ml (1m), 1495 ± 1941 EU/ml (156m), 1816 ± 1291 EU/ml (295m) and 2249 ± 721.5 EU/ml (empty control filter), respectively (Figure 1H).

### 3.2 BLG can be detected in bovine urine samples

**FIGURE 2:**
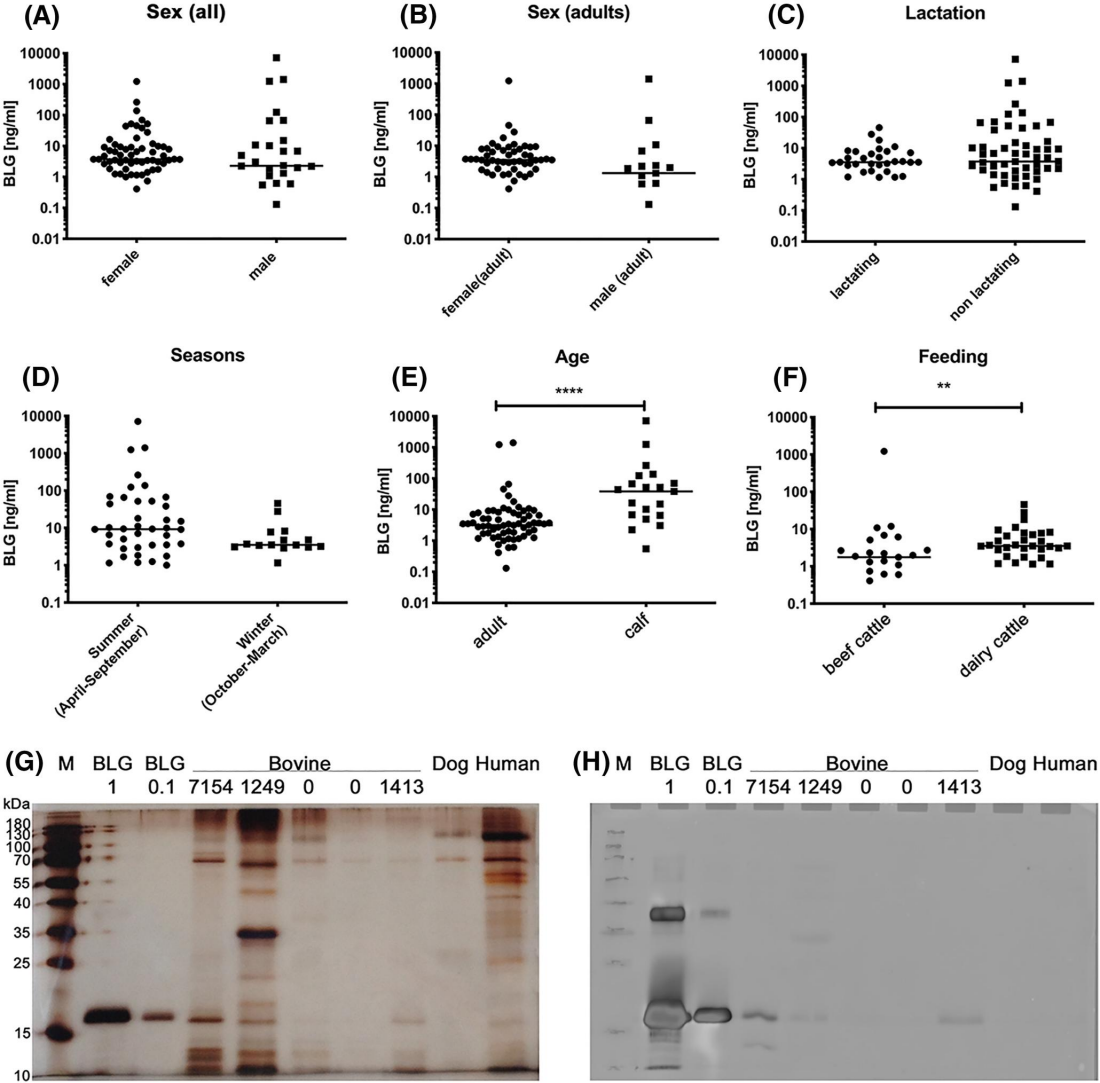
BLG in bovine urine samples, revealed by anti‐bovine BLG ELISA (A–F), silver stain (G), and immunoblot (H). BLG content of urine samples of cattle (*n* = 91; undiluted) from different stables determined by ELISA. Statistical comparison between (A) sexes among all animals, (B) sexes between adult animals, (C) lactation status of all animals, (D) season of urine sample collection (*n* = 57), (E) age of all animals, and (F) feeding conditions of beef cattle and dairy cattle (*n* = 57). Individual results are dotted on logarithmic scale on *y*‐axis, median is indicated. Indication: **p < 0.01, ****p < 0.0001; no indication: not significantly different. Presence of BLG was confirmed in (G) silver staining and (H) BLG‐specific immunoblot in selected urine samples (BLG values from ELISA indicated in ng/ml). BLG: commercial BLG at 1 and 0.1 μg/lane

Bovine urine samples (Table S2) were tested in ELISA to reveal the source of BLG in the stables. BLG was detectable at 0.5–1000 ng/ml (mean of all: 5 ng/ml) in urine samples of female and also male cattle. Considering the mean urine secretion of a cow of 40 L/d, this would reflect 6 mg/cow/month potentially aerosolized or distributed into dust. The statistical comparison revealed that the amount of BLG in urine was independent of sex, lactation status and season of urine collection (Figure 2A–D). Significantly higher BLG levels were found in urine of younger animals than adults (Figure 2E) and in dairy cattle versus beef cattle (Figure 2F). BLG in urine was confirmed in Coomassie (data not shown) and silver staining (Figure 2G) as well as in BLG‐specific immunoblot of selected samples (Figure 2H), where canine and human urine remained negative.

### 3.3 BLG is transferred into households and detected in bed dust samples

BLG levels were significantly higher in bed dust of cattle farms than in bed dust of poultry farms and urban apartments (Table S3), tested by BLG‐specific ELISA (Figure 1I).

### 3.4 BLG in stable and bed dust is associated with zinc

**FIGURE 3:**
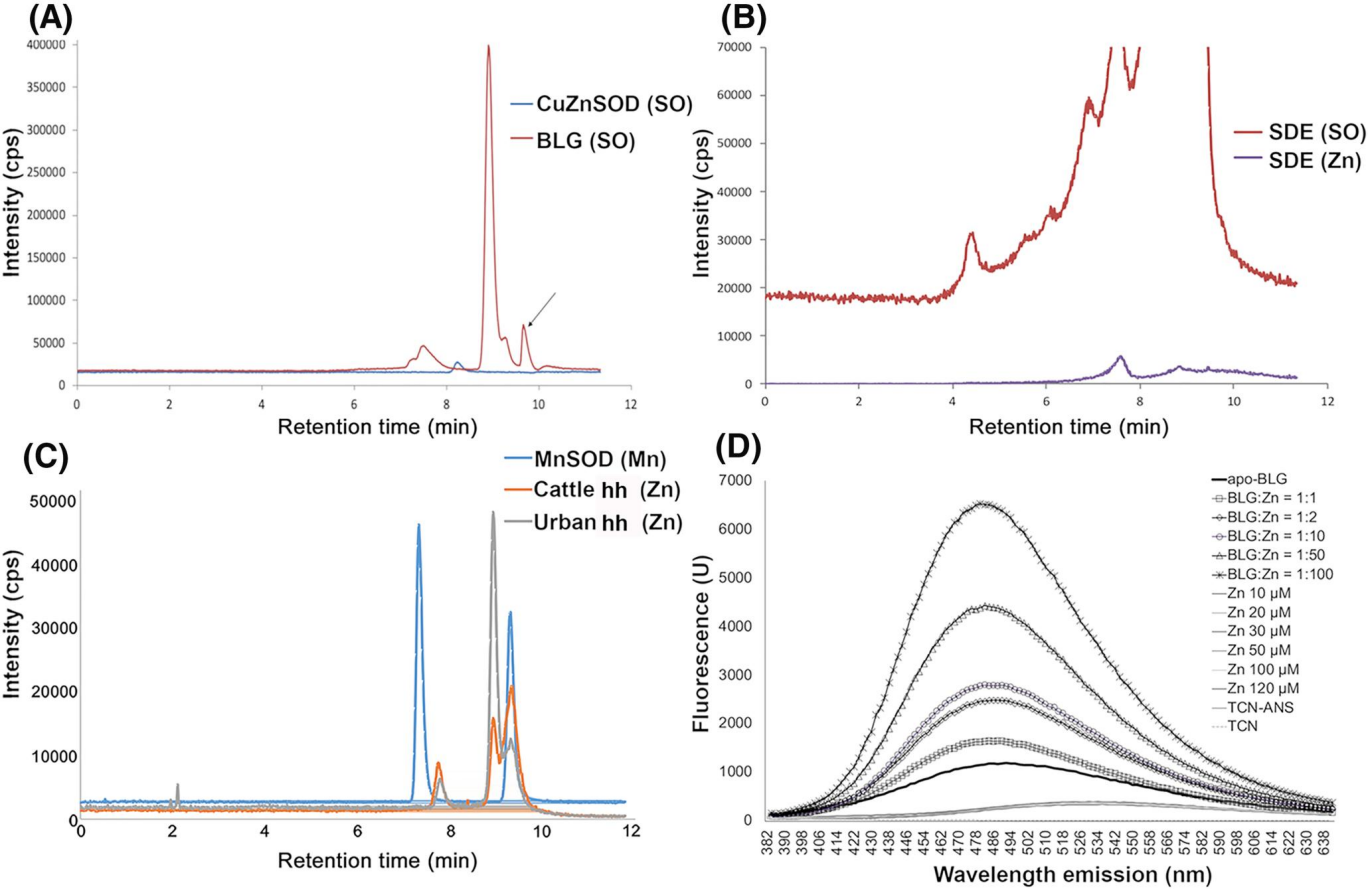
SEC‐ICP‐MS of dust samples. A, Commercially available BLG was used as reference (BLG), monomer peak at 9 min (18 kDa) and dimer at 7.8 min (36 kDa) retention time. Copper zinc superoxid dismutase (CuZnSOD) was used as size marker (dimer 32.5 kDa). SOD = superoxid dismutase; SO = sulfur oxide representing protein presence. Arrow indicates the lower cut‐off limit. SEC‐ICP‐MS of (B) stable dust extract (SDE) of cattle farm, and (C) in mattress dust of respective cattle households (cattle hh) and urban household (urban hh). Manganese SOD (MnSOD) was used as size marker (39.5 kDa). D, ANS fluorescence measurement of zinc association to BLG. BLG was deprived of its binding partners (apo‐BLG, 50 μM) and then complexed with zinc at different concentrations. Representative result of 3 repetitions is shown. ANS: fluorescence molecular probe; TCN: buffer

SEC‐ICP‐MS showed that stable dust and bed dust extracts from cattle farms contained BLG, comparing with BLG standard, which eluted as 2 peaks according to monomer at 9 min and dimer at 7.8 min (Figure 3A). In both, cattle stable dust extract (Figure 3B) as well as in bed dust extract (Figure 3C), the zinc‐peak was associated to the dimer of BLG, eluted at 7.8 min.

The association of zinc to BLG was confirmed via ANS‐assay by fluorescence measurement, where addition of zinc to apo‐BLG increased the fluorescence signal in a concentration‐dependent manner, as due to slight unfolding of BLG by zinc, more ANS molecules might bind (Figure 3D).

### 3.5 Zinc is closely associated to a B‐cell epitope on BLG

**FIGURE 4:**
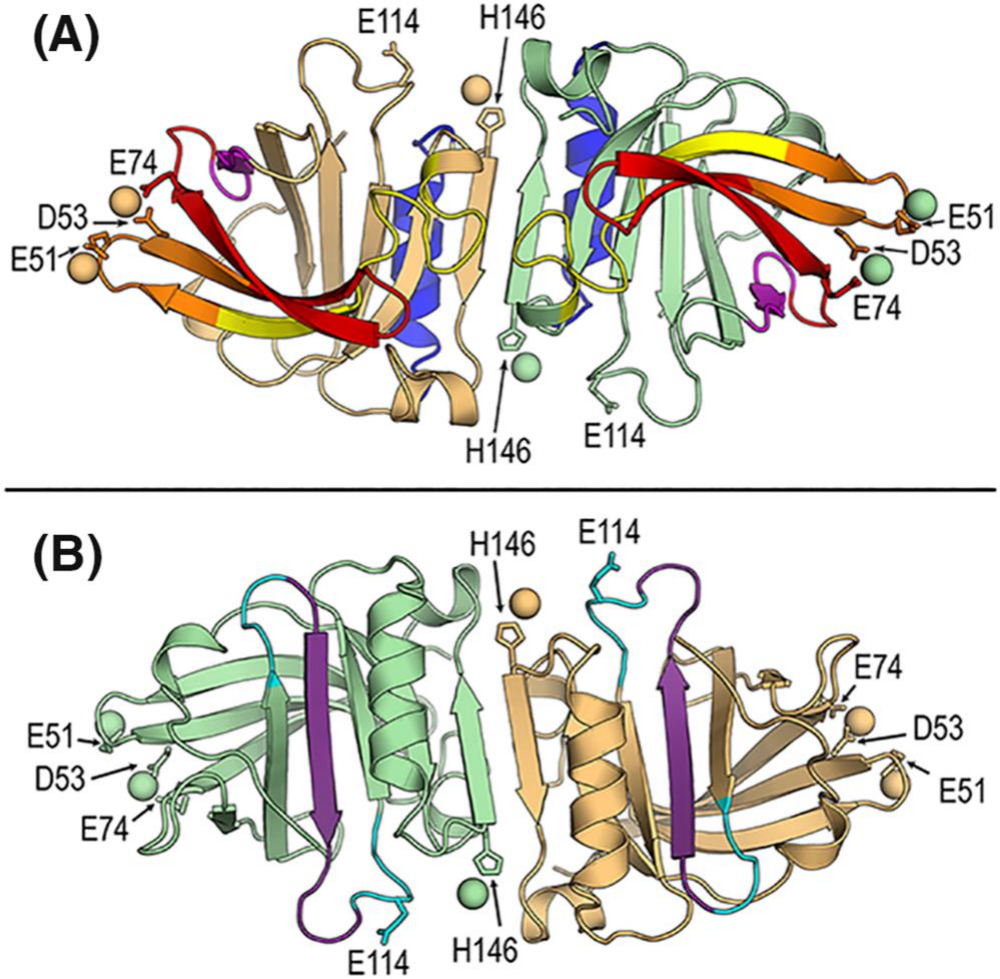
Structural localization of zinc on the BLG dimer related to B‐cell and T‐cell epitope regions. (A) B‐cell epitopes. Cartoon diagram of the crystal structure of dimeric BLG in the presence of 20 mM ZnCl_2_ (Protein Data Bank (PDB) id 4LZV). Zinc cations are depicted as spheres and residues coordinating their binding are labelled and shown as sticks. Residue E114 is also included because it participates in zinc‐binding in the BLG structure in the presence of 2 mM ZnCl_2_ (PDB id 4LZU). Color code for different B‐cell epitope regions: epitope 75–85 in magenta, epitope 127–144 in blue, weak stretch 1 (31–48) in yellow, weak stretch 2 (57–78) in orange, and weak stretch 3 (57–78) in red. (B) T‐cell epitope. Cartoon diagram as in (A) by 180° rotation around horizontal axis. T‐cell epitope 97–117 is colored cyan with the core segment 101–112 in violet

In epitope mapping,^20^, ^21^, ^22^, ^23^ one zinc ion near residue E74 was found to be very closely situated to the B‐cell epitope 75–85, with the distance from its nearest carboxyl oxygen to zinc being only 2.019 Å (Figure 4A). A weaker B‐cell epitope stretch 47–60 includes two of the three acidic residues coordinating two zinc cations, and the addition of a further weak stretch 57–78 covers the three acidic residues per BLG‐molecule. No zinc cation was found to be associated to a T‐cell epitope on BLG (Figure 4B).

### 3.6 Association of BLG with zinc induces a predominant Th1‐response in vitro

Apo‐ and zinc‐BLG or zinc alone were used to stimulate PBMC of healthy human donors. Endotoxin levels were comparable in both samples (apo‐BLG: 132.0 EU/ml, zinc‐BLG: 157.5 EU/ml). The efficacy of the zinc‐unloading method to generate apo‐BLG, and of zinc‐loading to generate zinc‐BLG was verified by flame atomic absorption spectroscopy (Figure S1).

**FIGURE 5:**
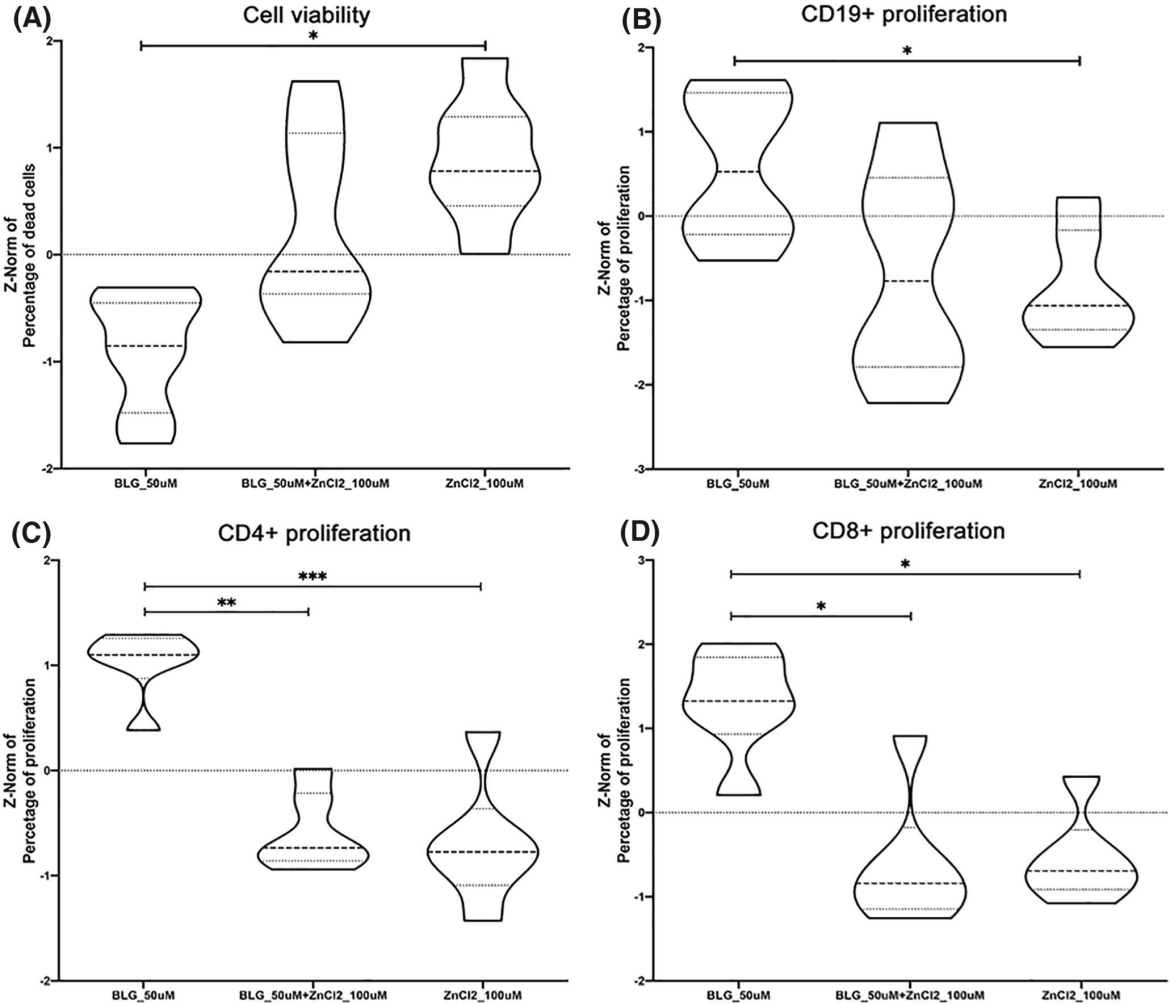
Cellular responses to BLG alone, zinc complexed with BLG, or zinc alone, studying PHA‐stimulated PBMC from healthy donors (*n* = 6). (A) Cell viability after stimulation with apo‐BLG (BLG_50 μM), zinc‐BLG (BLG_50 μM + zinc chloride (ZnCl2)_100 μM) or ZnCl2 (100 μM). Proliferation of PHA‐stimulated (B) CD19+, (C) CD3+CD4+ cells and (D) CD3+CD8+ cells. Mean of three independent experiments is shown. Statistical comparison of z‐normalized data was performed between three stimulation conditions (medium control was not different from apo‐BLG, data not shown): *p < 0.05, **<*p* < 0.01, ***p < 0.001. Representation as violin plots (wider parts represent values occurring at higher frequency; narrower parts represent values occurring at lower frequency; dotted line represents median, grey lines represent interquartile ranges)

Cell viability was affected by zinc (Figure 5A), while this was slightly counter‐balanced by addition of BLG. Gating on living cells, apo‐BLG (without zinc) had no influence on the proliferation of PHA‐stimulated cells compared to medium (data not shown). However, zinc alone resulted in a significantly lower PHA‐induced proliferation in CD19+ (Figure 5B), and additionally in association with BLG reduced proliferation of CD4+ cells (Figure 5C) and CD8^+^ cells (Figure 5D).

In cells without PHA‐stimulation, zinc on BLG (but not zinc alone) reduced the number of CD14^+^ cells among PBMC in a concentration‐dependent manner (Figure S2A); within the CD14^+^ cell population, CD4‐expression was lower when increasing zinc‐concentrations were offered together with BLG (Figure S2B).

**FIGURE 6:**
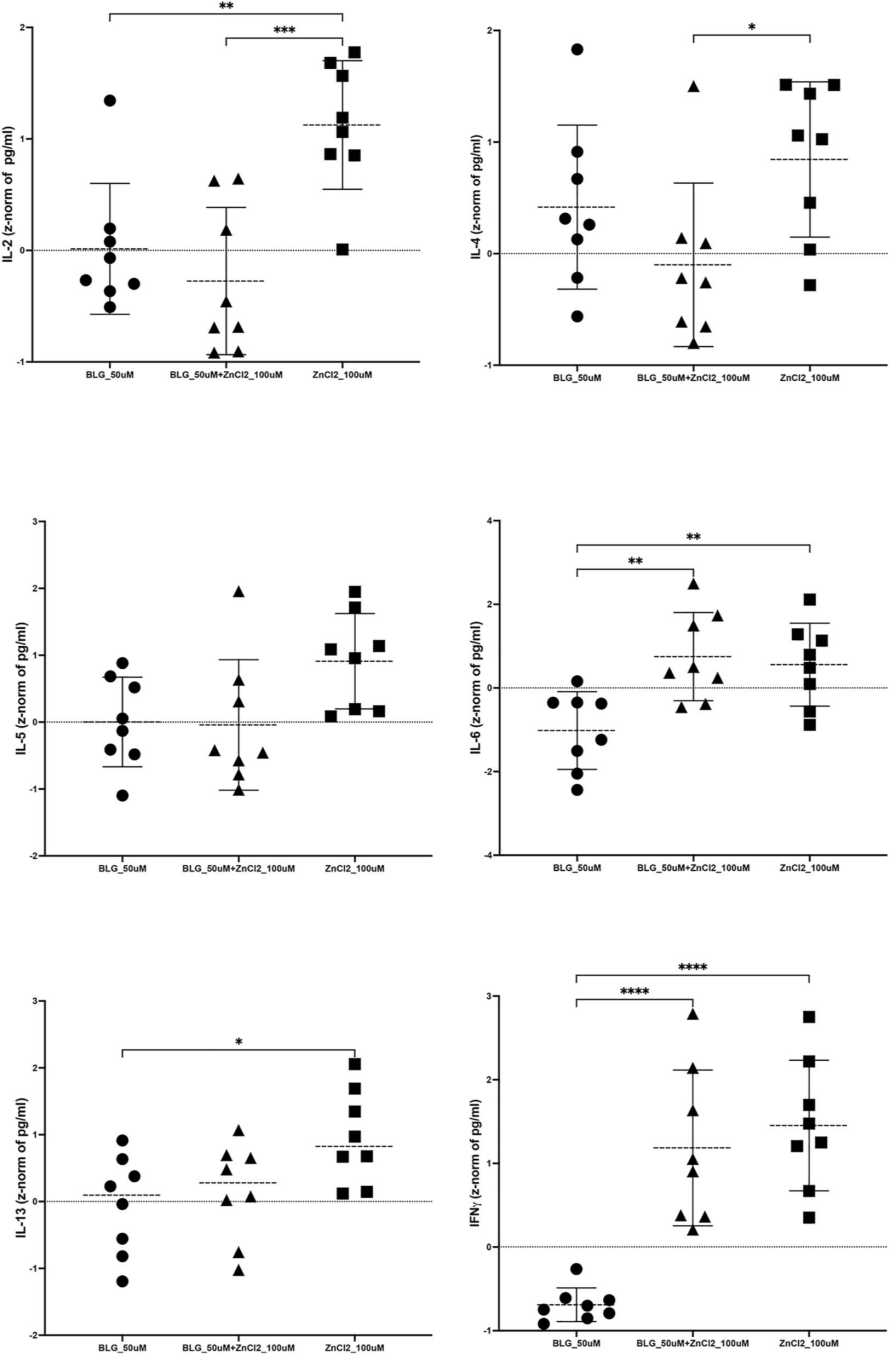
Cytokines in supernatants of PHA‐stimulated PBMC from healthy donors (*n* = 4), after incubation with apo‐BLG (BLG_50 μM), zinc‐BLG (BLG_50 μM + zinc chloride (ZnCl2)100 μM) or zinc chloride (ZnCl2_100 μM), were examined for (A) IL‐2, (B) IL‐4, (C) IL‐5, (D) IL‐6, (E) IL‐13 and (F) IFN‐γ by multiplexing in FACS. Statistical comparison of z‐normalized data was performed between three stimulation conditions (medium control was not different from apo‐BLG, data not shown): *p < 0.05, **p < 0.01, ***p < 0.001, ****p > 0.0001

Incubation of PHA‐stimulated PBMC with zinc‐BLG compared to apo‐BLG resulted in significantly higher levels of IL‐6 and especially IFN‐γ in supernatants of PBMC. No significantly higher induction of IL‐2, IL‐4, IL‐5, or IL‐13 was observed with zinc‐BLG compared to apo‐BLG (Figure 6). When zinc alone was applied to PHA‐stimulated PBMC, in addition to IFN‐γ and IL‐6, also high levels of cytokines IL‐2, IL‐4, IL‐5 and IL‐13 were released, diminishing the Th1‐dominance in the cytokine pattern (Figure 6). Levels of TNF‐α and IL‐10 did not significantly differ between the different stimulation conditions (data not shown).

### 3.7 Stable dust extract with BLG prevents allergy symptoms in mice

To confirm BLG as the active, functional component in the allergy‐protective cattle farm effect, we conducted an *in vivo* proof‐of‐concept study. Animals were pre‐treated i.n. with SDE+ (not depleted of BLG), SDE‐ (depleted of BLG), or water before i.p. double‐sensitization with Bet v 1 and BLG together with Al(OH)_3_ as Th2‐adjuvant (Figure S3A).

SDE+ and SDE‐ aliquots were also tested for endotoxin content, showing high amounts in both of them: 48,510 EU/ml in SDE+ and 109,900 EU/ml in SDE‐.

We aimed here to test whether the pre‐exposure to SDE+ has an impact on sensitization (i) in an antigen‐unspecific way by using the unrelated allergen Bet v 1 from birch pollen, or (ii) in an antigen‐specific manner using BLG itself for sensitization. Challenge with Bet v 1 in the double sensitized mice resulted in significantly lower symptom scores in the group pre‐treated with SDE+ than groups treated with SDE‐ or water (Figure S3B). Hence, SDE+ in a nonspecific manner protected against sensitization to an independent allergen. A similar trend could be observed after BLG‐challenge (n.s.; Figure S3B). For Bet v 1‐ and BLG‐specific IgE, lower levels were found in the SDE+ pre‐treated animals, paralleled by higher amounts of BLG‐specific IgG2a, resulting in the highest IgG2a/IgE‐ratios for Bet v 1 as well as for BLG in the SDE+ group (Figure S3C). This was accompanied by highest Th1‐cytokine IFN‐γ parallel to low IL‐6 levels in supernatants of BLG‐stimulated splenocytes of the SDE+ pretreated group (Figure S3D). However, differences were not statistically significant due to our small group size in this pilot experiment. No significant differences were seen in total IgE, specific Bet v 1‐ or BLG‐specific IgG1, or BLG‐specific IgA; Bet v 1‐specific IgG2a or IgA did not increase upon i.p. sensitization (data not shown).

## 4 DISCUSSION

In addition to microbes and their products, specific proteins may also play a role in the protective effect of farms against asthma, atopic eczema and allergies.^24^ Additionally, the fact that raw milk consumption *per se* is an independent protective factor,^25^ and especially the whey protein fraction seems to be important^26^ with 50% of whey proteins constituted by BLG, led us to the assumption that this protein could play an additional role here. Although BLG's cognate matrix is milk, we demonstrate here that it is also secreted in large amounts via cattle urine, explaining its delivery into stable dust. Using poultry stable dust as control, we could show that BLG indeed was specifically present in cattle farm samples, associated to zinc. In addition to BLG, endotoxin was found at higher levels in stable dust of cattle than poultry farms.^27^ This might be due to a specific microbiota composition in the different stables. In a recent paper, the microbiome of farm home dust was shown to contain cattle‐associated microbes that were typically absent from non‐farm households.^5^ However, even though BLG is a well‐known carrier for endotoxin, we found no statistically significant correlation between BLG and endotoxin levels.

In previous studies the limits for the protective farm effect were set within a distance below 390 m from the nearest cattle stable.^7^ We show here that also the concentration of aerosolized BLG declines steadily in a distance‐dependent gradient from the cattle stable, again implying a potential contribution of BLG to the farm effect. As expected, endotoxin levels were highest close to the stable. While BLG levels gradually declined with increasing distance to the stable, endotoxin declined more rapidly outside the stable. As BLG is a known endotoxin‐carrier, it was important to dissect the impact of endotoxin and zinc‐BLG. *In vitro*, PBMC were stimulated with apo‐BLG or zinc‐BLG, with both samples containing similar endotoxin levels. Also in our preliminary *in vivo* mouse model the applied SDE+ and SDE‐ both contained high endotoxin levels. Interestingly, SDE‐ contained even higher levels of endotoxin, probably due to more handling steps during depletion. These results show that differences *in vitro* and *in vivo* were independent of endotoxin levels, and therefore favour BLG as likely contributor to the protective farm effect.

The amounts of BLG were put in relation to the mean pulmonary capacity. Certainly, the composition of ambient air with different sizes of dust particles also impacts the pulmonary distribution of the inhaled particles, meaning that smaller carrier particles of BLG in the inhaled air could enter deeper lung compartments. We calculated the mean lung capacity without considering a potential difference in the uptake site for particles and/or BLG as such.

Urine was revealed as the major source of BLG in cattle stable dust. The concentration of BLG was higher in young animals and in dairy cattle, however, it was independent of sex, lactation status, season as well as breed. Interestingly, the human equivalent to BLG, human lipocalin‐2 (huLCN2), is used as a diagnostic urinary marker in chronic kidney disease^28^; but LCN2 also has a functional role: in cancer, where immune tolerance is detrimental, elevated huLCN2 levels are prognostic^29^, ^30^; in contrast, its levels are reduced in allergics and can be corrected by allergen immunotherapy, pointing towards a role in immune tolerance.^31^ Our novel collected evidence suggests that the lipocalin BLG might act tolerogenic also via aerosols.

As not all family members of a farm may be working or staying routinely and regularly in the stable, it was important to clarify the presence of BLG in the household. Even non‐farm homes can be protective when a farm‐like microbial environment is present in the home.^5^

We thus collected dust from cattle and poultry stables and corresponding mattress and pillow dust from farms and, for comparison, bed dust samples from urban apartments. There were significantly higher levels of BLG in dust of cattle farm households, compared to poultry farm or urban samples. Higher BLG levels in bed dust compared to respective stable dust are presumably the result of relatively higher protein concentration per weight of dust due to increased accumulation by vacuum‐cleaning. Moreover, bed dust contains less contaminants than the stable environment, where higher levels of non‐proteinic material are present, for example straw, hay, fodder, fine stone‐dust etc. In correlation with higher BLG concentrations at cattle farms, we recorded that relatively lower numbers of family members living on cattle farms reported to be affected by allergy (14.3%) compared to poultry farms (25%) or urban flats (60%). There was no clear correlation for BLG content in households of cattle farmers and their life‐style and habits (hair washing, mattress cleaning, pets in house), or number of animals on farms, again probably due to the low sample number. We also compared the farms with highest (*n* = 2) and lowest (*n* = 1) indoor‐BLG levels, but could not find obvious differences in behavior of farmers or other conditions, except for the fact that the household with lowest BLG in bed dust (but not in the stable) also had the lowest number of cattle (*n* = 1). Also for BLG in city apartments, no characteristic could explain high levels in bed dust, for example eating in bedroom, pets in household, type of mattress, or frequency of washing mattress or hair. Still, another group observed that newer mattresses contain more food allergens, and that a longer distance from kitchen to bedroom, the size of the dwelling, and cleaning equipment influenced the occurrence of food allergens in bed dust, incl. milk allergens.^32^

BLG, belonging to the lipocalin protein family, harbors an intramolecular pocket for small molecules or elements. This is relevant as in our previous studies we have shown that proteins from the lipocalin family modulate the immune response depending on their loading state: when the cavity is filled with compounds like siderophore‐iron^13^, ^33^, ^34^ or vitamin A,^11^, ^35^ lipocalins like BLG act tolerogenic, and an allergic immune response is avoided. Our SEC‐ICP‐MS analysis showed that the trace element zinc is associated to BLG in dust of stables and households. In contrast to the forementioned binding partners, zinc is not found within the calyx of BLG, but rather attached to the protein surface, which was also confirmed in the ANS‐assay, in analogy to crystallization studies.^11^, ^15^, ^35^ Zinc can induce a slight unfolding of BLG,^36^ giving access to more binding sites for ANS, seen as increasing fluorescence signal in the ANS‐assay.

A previously determined zinc‐BLG affinity constant of K_a = 1.5*10^5^ (1/M)^37^ and dissociation constant of K_d = 1/(K_a) = 6.7*10^−6^ M (i.e. 6.7 µMolar) indicate a moderate binding strength of zinc to BLG. When we mapped zinc on the predicted B‐ and T‐cell epitopes on a BLG‐dimeric molecule, we found the zinc ion near residue E74 very close to the B‐cell IgE‐epitope 75–85,^20^, ^21^, ^22^, ^23^ with the distance from its nearest carboxyl oxygen to zinc being only 2.019 Å. A weaker B‐cell IgE‐epitope stretch 47–60 includes two of the three acidic residues coordinating two zinc cations, and the addition of a further weak stretch 57–78 covers the three acidic residues. As a consequence, the presence of zinc cations on BLG could affect interactions with other molecules, including antibodies, or *vice versa*. Consequently, the immune recognition of BLG by B‐cells as non‐professional antigen‐presenting cells with the B‐cell receptor could be affected by the binding of zinc to BLG.

Complexing of zinc with BLG had a substantial influence on immune cells *in vitro* and decreased both, the proliferation of CD4+ as well as CD8+ T‐cells in a concentration‐dependent manner. These results indicate that addition of zinc, converting apo‐BLG into zinc‐BLG, can prevent the activation/proliferation of immune cells, probably also down‐regulating or preventing an inflammatory response, which we recently termed immune resilience.^13^ Antigen presentation by B‐cells could be affected as zinc ions might hinder binding and thus presentation to T‐cells in general. BLG complexed with zinc also induced a favorable Th1‐dominated pattern with high IFN‐γ levels versus low IL‐4, IL‐5 or IL‐13 levels, while zinc alone induced Th2‐cytokines IL‐4 and IL‐13 in pre‐stimulated PBMC of healthy donors, as also previously reported by others.^38^ Despite lowered proliferation of CD4+ and CD8+ cells by zinc‐BLG, a Th1‐response was promoted by this stimulant, which may be attributed to innate cells rather than T‐cells. In particular IL‐6 and IFN‐γ were associated with zinc‐BLG stimulation. IL‐6 is predominantly secreted by macrophages, whereas IFN‐γ is predominantly secreted by NK cells, making them the likely source for the Th1‐bias. Hence, zinc associated to BLG may add to the protective farm effect in an innate manner via Th1‐cytokine induction. Such Th1‐skewing was also described in human and animal studies.^39^, ^40^, ^41^ Regarding these Th1‐associated cytokines, we are also aware of the potential effect of LPS in our system. Simultaneously, we acknowledge that (i) complete LPS removal is hard to achieve without removing BLG from the sample itself, and (ii) BLG is an important natural carrier of LPS. Therefore, we measured endotoxin in our stimulation samples and could prove that they are similar in LPS‐content. We propose that this confirms that differences in proliferation and/or cytokine release in apo‐BLG versus zinc‐BLG were not due to LPS.

The proof‐of‐concept experiments in mice here suggest that the protective capacity of stable dust is dependent on BLG as major carrier component, since stable dust extract, in which BLG was depleted, showed a reduced allergy‐protective capacity. Importantly, the protective effect was independent of the endotoxin‐levels present in the samples. Rather, in line with our recently published animal studies, this immunomodulatory effect was dependent on BLG fulfilling a shuttle function for micronutrients like iron‐siderophore complexes, vitamin A and D,^12^, ^13^ and zinc, targeting them to innate immune cells. Importantly, a dietary lozenge containing whey protein with iron‐quercetin, vitamin A, and zinc could reduce symptoms in human birch‐pollen and house dust mite allergic patients,^42^, ^43^ as well as in the mouse model.^10^

Our study has some limitations: even though BLG represents a major fraction among the whey proteins, we can only speculate whether BLG with its binding partners also represents the allergy‐protective factor associated with raw milk. However, the facts that (i) milk processing destroys the tertiary structure of heat‐labile BLG,^44^, ^45^ (ii) this results in a loss of associated molecules or elements, and (iii) processed milk loses its allergy‐protective effect, makes this concept very plausible. Finally, the factors which determine natural BLG secretion and loading with zinc, such as cattle feed, health status and animal welfare, need to be determined.

## 5 CONCLUSION

Our data show that the secretory protein BLG is not only a major whey protein but that it is secreted by bovine urine, aerosolized around cattle farms and finally precipitated as a major compound in cattle farm dust. We have also shown that BLG complexed with zinc is able to induce a favorable Th1‐milieu in PBMC of healthy donors. Stable dust containing BLG resulted in better protection against allergic sensitization in an allergen‐nonspecific manner than dust depleted of BLG *in vivo* in the mouse model. We thus propose that‐in addition to endotoxin and microbial load‐BLG with its binding partners has innate immunomodulatory potency and may represent an active component in the well‐known allergy‐protective effect of cattle farms.

## CONFLICT OF INTEREST

IPS and EJJ report personal fees from Bencard Allergie GmbH, Germany, outside the submitted work. EJJ, LFP and FRW are inventors of EP2894478; “LCN2 as a tool for allergy diagnostic and therapy”, EP 14150965.3, Year: 01/2014; US 14/204,570, owned by Biomedical International R+D GmbH, Vienna, Austria, the basis for the dietary lozenge immunoBON®. EJJ is shareholder in this company. Dr. von Mutius reports grants from German Centre for Lung Research (DZL), personal fees from Elsevier GmbH, personal fees from Georg Thieme Verlag, personal fees from Springer‐Verlag GmbH, personal fees from Elsevier Ltd., personal fees from Chinese University of Hongkong, personal fees from European Commission, personal fees from HIPP GmbH & Co. KG, personal fees from AstraZeneca, personal fees from Massachusetts Medical Society, personal fees from Böhringer Ingelheim International GmbH, from European Respiratory Society (ERS), personal fees from Universiteit Utrecht, Faculteit Diergeneeskunde, personal fees from Universität Salzburg, personal fees from Springer Medizin Verlag GmbH, personal fees from Japanese Society of Pediatric Allergy and Clinical Immunology (JSPACI), personal fees from Klinikum Rechts der Isar, Munich, personal fees from University of Colorado, personal fees from Paul‐Martini‐Stiftung, Berlin, personal fees from Imperial College London, personal fees from Verein zur Förderung der Pneumologie am Krankenhaus Großhansdorf e.V., personal fees from Pneumologie Development, personal fees from Mondial Congress & Events GmbH & Co. KG, personal fees from American Academy of Allergy, Asthma and Immunology , personal fees from Margaux Orange, personal fees from Volkswagen Stiftung, personal fees from Österreichische Gesellschaft für Allergologie und Immunologie, personal fees from OM Pharma S.A., personal fees from Hanson Wade Ltd., personal fees from iKOMM GmbH, personal fees from DSI Dansk Borneastma Cemter, personal fees from American Thoracic Society, personal fees from Universiteit Utrecht, Faculteit Betawetenschappen, outside the submitted work; In addition, Dr. von Mutius has a patent Patent LU101064 pending, a patent Patent EP2361632 with royalties paid to ProtectImmun, a patent Patent EP1411977 with royalties paid to ProtectImmun, a patent Patent EP1637147 with royalties paid to ProtectImmun, and a patent Patent EP1964570 licensed to ProtectImmun and Erika von Mutius is: Member of the EXPANSE (funded by European Commission) Scientific Advisory Board, Member of the BEAMS External Scientific Advisory Board (ESAB), Member of the Editorial Board of “The Journal of Allergy and Clinical Immunology: In Practice”, Member of the Scientific Advisory Board of the Children’s Respiratory and Environmental Workgroup (CREW), Member of the International Scientific & Societal Advisory Board (ISSAB) of Utrecht Life Sciences (ULS), University of Utrecht, Member of External Review Panel of the Faculty of Veterinary Science, University of Utrecht, Member of the Selection Committee for the Gottfried Wilhelm Leibniz Programme (DFG), Member of the International Advisory Board of Asthma UK Centre for Applied Research (AUKCAR), Member of the International Advisory Board of “The Lancet Respiratory Medicine”, Member of the Scientific Advisory Board of the CHILD (Canadian Healthy Infant Longitudinal Development) study, McMaster University, Hamilton, Canada. The other authors declare no relevant conflict of interest in relation to this publication.

## AUTHOR CONTRIBUTIONS

Isabella Pali‐Schöll and Erika Jensen‐Jarolim designed the experiments; Isabella Pali‐Schöll evaluated results and performed mouse experiments and statistical analysis; Rodolfo Bianchini designed, performed and evaluated all cell stimulation experiments and cytokines; Gerlinde Hofstetter, Stella Ahlers, Theresa Altemeier, Simona Winkler performed sample collection of stable dust, household dust, and urine, as well as dust extraction, immunoblots, ELISA; Gerlinde Hofstetter evaluated cytokines in ELISA; Sheriene Moussa Afify planned and prepared substances for cell stimulation and animal experiments, and performed animal experiments; Anna D. J. Korath and Christina Pranger prepared and characterized BLG‐depleted SDE for animal experiments; Hanna Mayerhofer performed PBMC experiments; Karin Hufnagl was responsible for patient recruitment and ethical approval, and helped with animal experiments; Raimund Widhalm analyzed samples for zinc binding in flame atomic absorption spectroscopy; Anne Kasper‐Giebl designed and helped with aerosol sampling; Luis F. Pacios performed the epitope mapping and provided affinity information; Thomas Wittek provided and helped with stable dust and urine sampling and critically discussed results and manuscript; Donata Vercelli and Erika von Mutius provided Bavarian stable dust and critically discussed and helped with manuscript preparation; Franziska Roth‐Walter helped with animal experiments, and together with Erika Jensen‐Jarolim supported data interpretation and critically edited the manuscript. Erika Jensen‐Jarolim conceived the study. All authors have read and approved the final version of the manuscript.

## Supporting information

Supplementary Material S1

Figure S1

Figure S2

Figure S3

Figure S4

## Data Availability

The data that support the findings of this study are available from the corresponding author upon reasonable request.
