# Supplementary material for: Secretory protein beta‐lactoglobulin in cattle stable dust may contribute to the allergy‐protective farm effect
Source: Clin Transl Allergy. 2022 Feb 12;12(2):e12125. doi: 10.1002/clt2.12125 (PMC8840802; doi:10.1002/clt2.12125)
Supplement: Supplementary file 1 — Supplementary Material S1 [file CLT2-12-e12125-s004.docx]

**Supplement**

*Pali-Schöll I et al.*  *BLG in stable dust*

**Secretory protein beta-lactoglobulin in cattle stable dust may contribute to the allergy-protective farm effect**

Isabella Pali-Schöll^1,2*^, Rodolfo Bianchini^1^, Sheriene Moussa Afify^1,3^, Gerlinde Hofstetter^1^, Simona Winkler^1^, Stella Ahlers^1^, Theresa Altemeier^1^, Hanna Mayerhofer^1^, Karin Hufnagl^1^, Anna D. J. Korath^1^, Christina Pranger^1,2^, Raimund Widhalm^4,5^, Stephan Hann^6^, Thomas Wittek^7^, Anne Kasper-Giebl^8^, Luis F. Pacios^9^, Franziska Roth-Walter^1,2^, Donata Vercelli^10^, Erika von Mutius^11^, Erika Jensen-Jarolim^1,2 *^

**Table S1:**Sets of sampling for dust and urine samples.

| **Set number**  Application | **Stable ID** | **Sampling method(s)** |
| --- | --- | --- |
| **Set 1** |  | |
| Extraction method;  Collection method | Bav | settling on cardboard box |
|  | N1, N2, T | wiping |
|  | Vet | settling on cardboard box,  wiping |
| Urine samples | N1, N2, T, Vet, W, K | urine collection  (further samples provided by AGES) |
| **Set 2** |  | |
| Species differences;  Household samples | C1-14 | wiping in stable, vacuum-cleaning in respective household |
|  | P1-8 | wiping in stable, vacuum-cleaning in respective household |
|  | U | vacuum-cleaning in household |
| **Set 3** |  | |
| Filter material | Vet | cellulose filter  quartz fiber filter  teflon filter |
| **Set 4** |  | |
| Distance measurement | Vet (distances 1, 156, 290m) | cellulose filter |

**Table S2:** Bovine urine samples (n=91) from cattle stables in Germany and Austria.

| **Stable ID** | **Lactation** | | | | **Sex** | | **Total number of samples** |
| --- | --- | --- | --- | --- | --- | --- | --- |
|  | Lactating | Non-lactating | | | Male | Female |  |
|  |  | NEL | Calf | Dry cow |  |  |  |
| N1 | 9 |  |  |  |  | 9 | 9 |
| N2 | 9 |  |  |  |  | 9 | 9 |
| T | 3 |  |  |  |  | 3 | 3 |
| V | 2 | 4 | 1 | 10 | 2 | 15 | 17 |
| W |  |  | 14 |  | 6 | 8 | 14 |
| K | 5 |  |  |  |  | 5 | 5 |
| A | 3 | 25 | 6 |  | 20 | 14 | 34 |
| Total | 31 | 29 | 21 | 10 | 28 | 63 | **91** |

Bovine urine samples (n=91) collected in Northern Germany (N1, N2), Tyrol/Austria (T), Vienna/Austria (V), Wels/Austria (W), Kremesberg/Austria (K), or provided by the Austrian Food Safety Agency AGES (A). NEL: never-ever lactating.

**Table S3:** Comparison of stable and household characteristics together with BLG concentration in dust collected from stables and beds of cattle farms, poultry farms, and urban apartments.

|  | BLG in **bed**  dust extract*  (ng/mg sieved dust) | BLG in **stable**  dust extract*  (ng/mg sieved dust) | Eating in bedroom | Number of cattle | Type of farm | Farming conditions | Type of stable | Family members affected by allergies or asthma;  N (type of allergy) |
| --- | --- | --- | --- | --- | --- | --- | --- | --- |
| C1 | 37,97 | 8,26 | no | 36 | dairy | open | loose-house | 0 |
| C2 | 10,52 | 3,49 | no | 100 | dairy | open | loose-house | 0 |
| C3 | 77,11 | 2,71 | no | 50 | dairy | open | loose-house | 0 |
| C4 | 46,84 | 3,80 | no | 40 | dairy | open | loose-house | 0 |
| C5 | 71,25 | 2,40 | no | 70 | dairy | open | loose-house | 0 |
| C6 | 9,60 | 3,22 | no | 40 | dairy | open | loose-house | 0 |
| C7 | 56,86 | 6,94 | no | 50 | dairy | open | loose-house | 1 (farmer’s lung) |
| C8 | 103,39 | 8,46 | no | 47 | dairy | closed | loose-house | 0 |
| C9 | 224,87 | 3,82 | no | 60 | dairy | open | loose-house | 0 |
| C10 | 80,81 | 1,19 | no | 60 | dairy | closed | loose-house | 1 (bee venom, insect venoms) |
| C11 | 240,17 | 3,01 | no | 90 | beef | closed | deep bedding, full slatted floor | 0 |
| C12 | 39,68 | 6,97 | n.i. | n.i. | beef | n.i. | n.i. | 0 |
| C13 | 13,46 | 1,26 | no | 26 | suckle herd | open | loose-house | 0 |
| C14 | 6,09 | 3,10 | no | 1 | dairy | open | loose-house | 0 |
| P1 | 8,22 | 1,99 | no | - | breed poultry | open | free range | 0 |
| P2 | 7,49 | 0,10 | no | - | egg | closed | barn farming | 1 (weed pollen, lime tree pollen) |
| P3 | 15,05 | 0,77 | no | - | egg and meat | open | mobile stable and free range | 0 |
| P4 | 3,82 | 0,03 | no | - | egg | open | mobile stable and free range | 1 (oranges) |
| P5 | 7,68 | 0,00 | no | - | egg | closed | barn farming | 0 |
| P6 | 38,00 | 0,55 | no | - | egg | closed | barn farming | 0 |
| P7 | 1,87 | 0,00 | no | - | meat | open | free range | 0 |
| P8 | 54,54 | 0,00 | yes |  | meat | closed | barn farming | 0 |
| U1 | 4,66 | - | no | - |  |  |  | 2 (contact allergy methyl-isothiazolinone; mosquito bites) |
| U2 | 35,78 | - | yes | - |  |  |  | 0 |
| U3 | 10,85 | - | yes | - |  |  |  | 0 |
| U4 | 6,59 | - | yes | - |  |  |  | 0 |
| U5 | 3,20 | - | no | - |  |  |  | 1 (allergic asthma to house dust mite, weed pollen, animal dander, plane tree pollen) |
| U6 | 36,63 | - | yes | - |  |  |  | 1 (house dust mites, weed pollen) |
| U7 | 24,66 | - | no | - |  |  |  | 0 |
| U8 | 17,09 | - | no | - |  |  |  | 1 (unknown; |
| U9 | 2,68 | - | no | - |  |  |  | 1 (weed and other pollen) |
| U10 | 12,50 | - | yes | - |  |  |  | 0 |

*Numbers for BLG in stable and bed dust extracts are given in ng/ml extract without decimal numbers. C = cattle household; P = poultry household; U = urban apartment; n.i. = no information; - = not applicable

**Table S4:**Description of the antibodies used for PBMC surface marker staining analysis in BLG w/wo zinc stimulation experiments.

| **Antibody** | **Clone** | **Fluorochrome** | **Provider** |
| --- | --- | --- | --- |
| anti-human mAb CD19 | SJ25C1 | PE/CY7-labeled | BioLegend, San Diego, CA |
| anti-human mAb CD14 | M5E2 | APC/CY7-labeled | BioLegend, San Diego, CA |
| anti-human mAb CD3 | SK7 | APC-labeled | BioLegend, San Diego, CA |
| anti-human mAb CD4 | SK3 | PE-labeled | BioLegend, San Diego, CA |
| mouse IgG1 isotype control mAb | MOPC-21 | PE/CY7-labeled | BioLegend, San Diego, CA |
| mouse IgG1 isotype control mAb | MOPC-21 | PE-labeled | BioLegend, San Diego, CA |
| mouse IgG1 isotype control mAb | MOPC-21 | APC-labeled | BioLegend, San Diego, CA |
| mouse IgG2a isotype control mAb | MOPC-173 | APC/CY7-labeled | BioLegend, San Diego, CA |

**Pali-Schöll et al., Supplementary Figure - Legends**

**Suppl. Figure 1:** Efficacy of preparation of apo-BLG and zinc-BLG controlled by flame atomic absorption spectroscopy. Apo-BLG = 50 µM BLG; zinc-BLG = 50 µM BLG+100 µM zinc chloride; ZnCl2 = 100 µM zinc chloride.

**Suppl. Figure 2:** Dose-dependent effects of zinc on CD4 expression in CD14 monocytes. PBMC of healthy donors (n=5), without pretreatment of PHA, were stimulated with apo-BLG, zinc-BLG with varying concentrations of zinc, or different concentrations of zinc, and investigated for expression of (**A**) CD14 and (**B**) CD4 within the CD14+ population.

**Suppl. Figure 3:** (**A**) Treatment scheme: female BALB/c mice (n=5) were pre-treated intranasally on 2 consecutive days with stable dust extract (SDE) containing BLG (SDE+), SDE depleted of BLG (SDE-) or water, in 5 cycles, followed by double sensitization with BLG + birch pollen allergen Bet v 1 (1:1) + alum i.p. twice. Allergen challenge was performed with BLG and Bet v 1 in the same animals on consecutive days before sacrifice. Blood samples were drawn as indicated in (A). (**B**) Symptom scores after allergen challenge. (**C**) Antigen-specific antibodies IgG2a/IgE ratios. * p≤0.05. (**D**) Cytokines IFN-γ, IL-5, IL-10 and IL-6 released from splenocytes stimulated for 72 hours with apo-BLG, apo-Bet v 1 or Con A (positive control); the individual values of medium-stimulation (background control) are subtracted.

**Suppl. Figure 4:** Gating strategy in FACS analysis for PBMC treated with PHA and sub-set analysis.

**Supplementary methods**

**Collection and extraction of dust samples from stables, bedrooms and environmental air around stables**

Dust samples (**Table S1, set 1**) from cattle stables were collected by settling (3 weeks on cartonnage) or wiping from elevated surfaces: Bavarian stable (stable n=1, 1 sample); 3 samples each were collected from stables in Northern Germany (stables n=2) and Austria (stables n=2) in July 2016, January 2017 and March 2017 for comparison of efficacy of collection and extraction methods. Dust samples were extracted and analyzed by ELISA and immunoblot.

Dust samples (**Table S1, set 2**) from dairy and beef cattle barns (n= 14) and poultry farms (n=8; without cattle on the same farm), all with traditional farming conditions, were collected by wiping between June and July 2018 in Lower Austria. All cattle farms had a similar farming type (**Table S3**), like animal numbers between 1-100, straw as bedding, manure removal (slatted floor or mechanical) and ventilation in the stable. At each of those farms, also dust samples from beds of the corresponding households were collected, and in addition samples from urban apartments (n=10; Vienna). Dust collection was performed by vacuum cleaning of mattresses (4 min) and pillows (1 min). Participants filled in a questionnaire about farm conditions and life-style (e.g. habits of hair washing, changing of clothes and bed cover, pets in household).

Ambient aerosols (total suspended particles) were collected for pre-experiments to investigate suitability of sampling material (**Table S1, set 3**) on cellulose filters (Whatman, grade 40, 47 mm, No 1440-047), quartz fiber filters (Pall, Tissuquartz-2500QAT-UP) or teflon filters (Zeflour, supported PTFE, Pall Life Science (P/N P5PL047)). As all carrier materials allowed good retrieval of proteins (data not shown), further sampling was performed on the easy-to-handle cellulose filters. Sampling time of ambient aerosols during final measurements (**Table S1, set 4**) was 5-6 days, at 0 m, 1 m, 156 m and 290 m distance from the stable. Control samples were used from a previous experimental setting, taken at a high alpine background site (Sonnblick Observatory, 3106 m above sea level) using quartz fiber filters. In this case sampling time was one week, sampling was carried out in cooperation with ZAMG (Zentralanstalt für Meterologie und Geodynamik).

Wiped dust samples were collected and pooled within on stable (height: 2-4 m, e.g. from window sills and pipe systems). Settled dust was collected on one cardboard box/stable (height: 2-4 m) for 3 weeks. Ambient aerosols were collected with the filter head mounted in the stable (height of 2-4 m) or on buildings outdoors for distance measurement.

**BLG-detection in dust of stables and air samples, and in urine samples**

BLG-specific immunoblots were performed with two different antibody combinations acc. to sensitivity after pre-experiments: in dust samples, polyclonal rabbit anti-bovine-lactoglobulin, Genway Biotech, GWB-334CF4 and polyclonal goat anti-rabbit IgG (H+L)-HRP, Jackson Immuno Research, No. 111-035-003 were used; for air samples: abcam, ab112893 and abcam ab205718 were applied, all according to manufacturer’s instructions.

In immunoblot, reaction was induced by ECL substrate (clarity TM Western ECL Substrate, BioRad, #170-5061) and luminescence was detected by continuous detection with the ChemiDoc™Touch Imaging System (BioRad) at the chemiluminescence mode.

**Endotoxin-detection in dust samples of cattle and poultry farms**

Endotoxin was detected by a homogenous fluorescence-based microplate assay with the recombinant factor C (rFC) (Endozyme®). Factor C is the first endotoxin-specific receptor of the LAL-cascade and an essential part of EndoZyme® assay. After activation by endotoxin, the rFC cleaves a synthetic substrate, which results in a fluorescence signal, measured thereafter in a TECAN® reader.

**Mass spectrometry**

For sample preparation, bands of interest were excised manually from Roti-blue stained 1D gels. After washing and destaining^1^, spots were reduced with dithiothreitol and alkylated with iodoacetamide^2^. In-gel digestion was performed with trypsin (Trypsin Gold, Mass Spectrometry Grade, Promega, Madison, WI) with a final trypsin concentration of 20 ng/µl in 50 mM aqueous ammonium bicarbonate and 5 mM CaCl_2_. Digest proceeded for 8 hours at 37°C^3^. Afterwards, peptides were extracted with three changes of 30 µL of 5% trifluoro acidic acid in 50% aqueous acetonitrile supported by ultrasonication for 10 min per change. Extracted peptides were dried down in a vacuum concentrator (Eppendorf, Hamburg, Germany). LC-MS/MS analysis: Peptides were separated on an Eksigent micro-LC system (Sciex). Sample pre-concentration and desalting was accomplished with a 5 x 0.5 mm trap column (YMC). For sample loading and desalting 2% ACN in ultra-pure H_2_O with 0.1% FA was used as a mobile phase with a flow rate of 10 µl/min. Separation of peptides was performed on a YMC Triart C18 3um 150 x 0.3mm column with a flow rate of 5µl/min. The gradient started with 5% B (100% ACN with 0.1% formic acid) and increased to 25% B in 68 min and to 35% in 5 min. It was followed by a washing step with 80% B. Mobile Phase A consisted of ultra-pure H_2_O with 0.1% formic acid. For mass spectrometric analysis the LC was directly coupled to a high-resolution quadrupole time of flight mass spectrometer (Triple TOF 5600, Sciex). For information dependent data acquisition (IDA runs) MS1 spectra were collected in the range of 350 - 1500 m/z. The 20 most intense precursors with charge state 2–4, which exceeded 250 counts per second, were selected for fragmentation for 250 ms. MS2 spectra were collected in the range 100–1600 m/z for 75 ms. Precursor ions were dynamically excluded from reselection for 10 s. The database for protein identification was downloaded from the publicly available UniProt database (www.uniprot.org).

**Zinc measurement by flame atomic absorption spectroscopy**

Lysates were acid-digested with nitric acid (69%; Suprapur®, Carl Roth) in a microwave oven (MARS6, CEM Corporation) and analyzed by flame atomic absorption spectroscopy (Zeenit 700P, Analytik Jena). Reference material (Seronorm Whole-Blood L-2 (LOT 1702858) and Seronorm Urine L-2 (LOT 1706878)) had zinc levels (6.3 mg/l ± 0.3 and 1.2 mg/l ± 0.01) that lay within the certified ranges (4.8-6.8 mg/l and 0.99-1.37 mg/l). The limit of detection (LOD) was 0.0026 mg/L. All samples were measured in duplicate by the working curve method (RSD<15%).

**Cell stimulation experiments and flow cytometry analysis**

PBMCsPBMC were obtained from healthy donors using Ficoll (1.077 g/L) purification. Total PBMC were counted and diluted at a concentration of 1x10^7^ cells/ml before staining with CFSE (5 μM final concentration). CFSE-labeled cells were seeded in a 96-well cell culture flat bottom plate at the concentration of 1.5x10^6^ cells/ml (100 μl volume/well).

Apo-BLG and ZnCl_2_ were prepared freshly for every experiment and sterilized using 0.22 μm syringe filters. The stock concentration of apo-BLG was 500 μM; the stock concentration of ZnCl_2_ was 500, 1000, 1500 μM. Apo-BLG with or w/o ZnCl_2_ were mixed to form zinc-BLG (1:1, 1:2, 1:3 with ZnCl_2_; ; ratios derived from crystallization studies from https://www.rcsb.org/structure/4LZU and https://www.rcsb.org/structure/4LZV). From apo-BLG or ZnCl_2_, 20μl (at different concentrations) or of zinc-BLG (1:1, 1:2, 1:3 with ZnCl_2_) were distributed in each well (1:10 dilution of stock concentration). Cells were treated or not with Phytohaemagglutinin (PHA)-L (L4144 Sigma) at a final concentration of 4μg/ml. After 48h of stimulation, cells were detached using D-PBS (without Ca^2+^ and Mg^2+^) plus EDTA 2.5mM.

Specific staining: cells were stained with anti-CD19 PE-Cy7 (Mouse IgG1-k, clone SJ25C1, cat n. 363012, BioLegend) for B-cells; anti-CD4 PE (Mouse IgG1-k, clone SK3, cat n. 344606, BioLegend) for CD4 helper T-cells and monocytes; anti-CD3 APC (Mouse IgG1-k, clone SK7, cat n. 344812, BioLegend) for T-cells; anti-CD14 APC-Cy7 (Mouse IgG2a-k, clone M5E2, cat n. 301820, BioLegend) for monocytes. Isotype controls: Mouse IgG1-k PE-Cy7 (clone MOPC-21, cat n. 400126, BioLegend); Mouse IgG1-k PE (clone MOPC-21, cat n. 400112, BioLegend); Mouse IgG1-k APC (clone MOPC-21, cat n. 400119, BioLegend); Mouse IgG2a-k APC-Cy7 (clone MOPC-173, cat n. 400230, BioLegend).

After 48h of stimulation, cells were detached using ice cold D-PBS (without Ca^2+^ and Mg^2+^) plus EDTA 2.5mM, and washed twice with HBSS before surface marker phenotypization. Then, the cells were incubated with reconstituted Zombie Violet™ (No 423113, BioLegend, California, USA) live/dead discrimination dye in accordance with manufacturer's recommendations. Briefly,  lyophilized Zombie Violet™ was reconstituted with RT pre-warmed DMSO. The cell pellets were incubated for 15 min at room temperature protected from light with 40 µl of reconstituted Zombie Violet™ diluted 1:500 in HBSS. After that and without any washing step, the cells were incubated with a multicolor staining mix of monoclonal antibodies (**Table S4**) against CD19 for B-cells, CD14 for monocytes, CD3 for total T-cells, and CD4 for helper T-cells or their isotype controls (BioLegend, San Diego, CA) diluted 1:100 with specific staining buffer. The staining buffer dilution volume was calculated by subtracting the reconstituted Zombie Violet™ volume already present in the staining tubes. The cells were incubated at RT for additional 30 min and washed twice with staining buffer (No420201, BioLegend, California, USA). The samples were acquired in FACS Canto II flow cytometers (Becton Dickinson, Franklin Lakes, NJ). Twenty-thousand events were recorded and analyzed with the FlowJo software version 10.7 (Becton Dickinson, Franklin Lakes, NJ). The FITC channel was used to determine the dilution of CFSE dye emission, which is directly correlated with an increase of  proliferation of the considered PBMC subpopulation. Statistical analysis was performed by using GraphPad Prism software version 9.1 (GraphPad Software LLC, California, USA).

**Flow cytometry analysis and gating strategies**

Samples were acquired with FACS Canto II flow cytometers (Becton Dickinson, Franklin Lakes, NJ). Twenty-tousand events were recorded and analyzed with the FlowJo software version 10.7 (Becton Dickinson, Franklin Lakes, NJ). Gating strategies are presented in Suppl Fig. 4. Statistical analysis was performed by using GraphPad Prism software version 9.1.

**Mouse model**

Symptoms were scored after i.p. allergen challenge with BLG and the day after with Bet v 1 (day of sacrifice from 0-5 (0, no symptoms; 1, scratching around nose and head; 2, puﬃness around eyes and mouth; 3, wheezing, labored respiration, cyanosis around mouth and tail; 4, no activity after prodding, or tremor and convulsion; 5, death) acc. to Li et al.^4^ by a blinded observer.

BLG and Bet v 1 specific IgG1, IgA, IgG2a and IgE levels in sera were measured by ELISA. Microtiter plates (Maxisorp, Nunc, Roskilde, Denmark) were coated (200 µl/well) with either BLG (10 µg/ml) or Bet v 1 (5 µg/ml) in carbonate coating buffer (pH 9.6) and incubated overnight at 4°C. Serum was added, diluted 1:100 for IgG1, IgA, IgG2a and 1:15 for IgE (100 µl/well) after washing and blocking with 1% BSA in PBS (RT/ 1 h) and incubated overnight at 4°C. Also, serial dilutions of mouse IgG1, IgG2a, IgE, and IgA standards were used. Monoclonal rat anti-mouse antibodies (eBiosciences), IgG1 (clone A85–1), IgG2a (clone R19-15), IgG2b (clone R12-3), IgA (clone c10-1), or IgE (clone R35-72) were applied, followed by incubation with polyclonal peroxidase-labeled goat anti-rat IgG antibodies (GE Healthcare). For total IgE, anti-mouse IgE (553413), standard mouse IgE (553413), biotin-rat-anti-mouse IgE (553419) and streptavidin-HRP (554066), all from BD Pharmingen, were used. Tetramethylbenzidine (eBiosciences) was used as substrate and 1.8 M sulfuric acid was used as stop solution followed by optical density measurement at 450 nm.

**Supplementary references**

1. Gharahdaghi F, Weinberg CR, Meagher DA, Imai BS, Mische SM. Mass spectrometric identification of proteins from silver-stained polyacrylamide gel: a method for the removal of silver ions to enhance sensitivity. Electrophoresis 1999; 20:601-5.

2. Jimenez CR, Huang L, Qiu Y, Burlingame AL. In-gel digestion of proteins for MALDI-MS fingerprint mapping. Curr Protoc Protein Sci 2001; Chapter 16:Unit 16 4.

3. Shevchenko A, Wilm M, Vorm O, Mann M. Mass spectrometric sequencing of proteins silver-stained polyacrylamide gels. Anal Chem 1996; 68:850-8.

4.  Li XM, Schofield BH, Huang CK, Kleiner GI, Sampson HA. A murine model of IgE-mediated cow's milk hypersensitivity. J Allergy Clin Immunol 1999; 103:206-14.
