# Supplementary figures and images for: Secretory protein beta‐lactoglobulin in cattle stable dust may contribute to the allergy‐protective farm effect
Source: Clin Transl Allergy. 2022 Feb 12;12(2):e12125. doi: 10.1002/clt2.12125 (PMC8840802; doi:10.1002/clt2.12125)

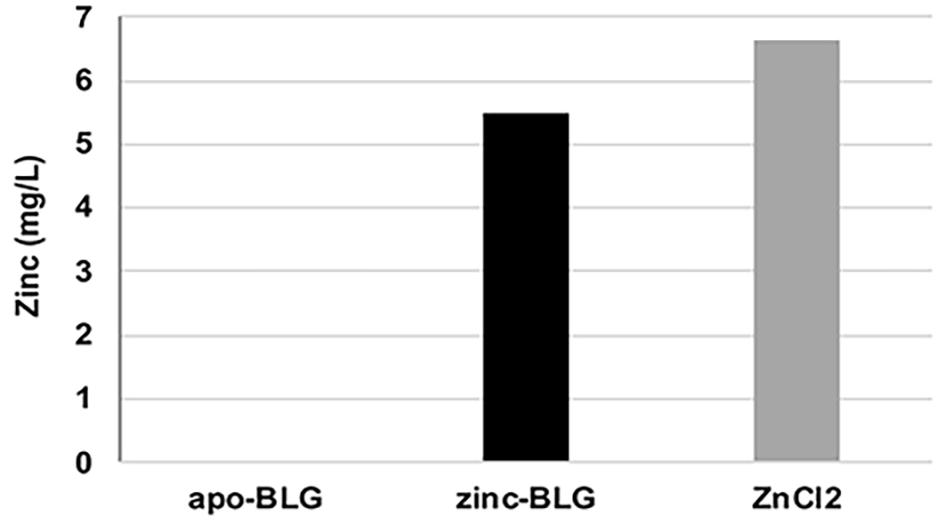

Supplement: Supplementary file 2 — Figure S1 [file CLT2-12-e12125-s001.tif]

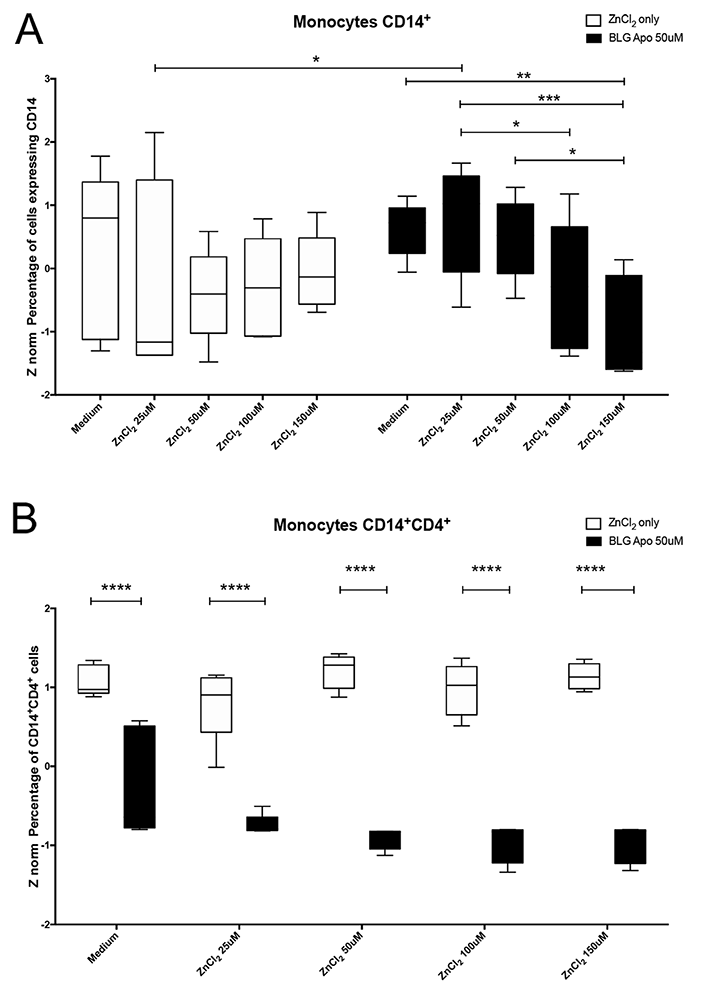

Supplement: Supplementary file 3 — Figure S2 [file CLT2-12-e12125-s002.tif]

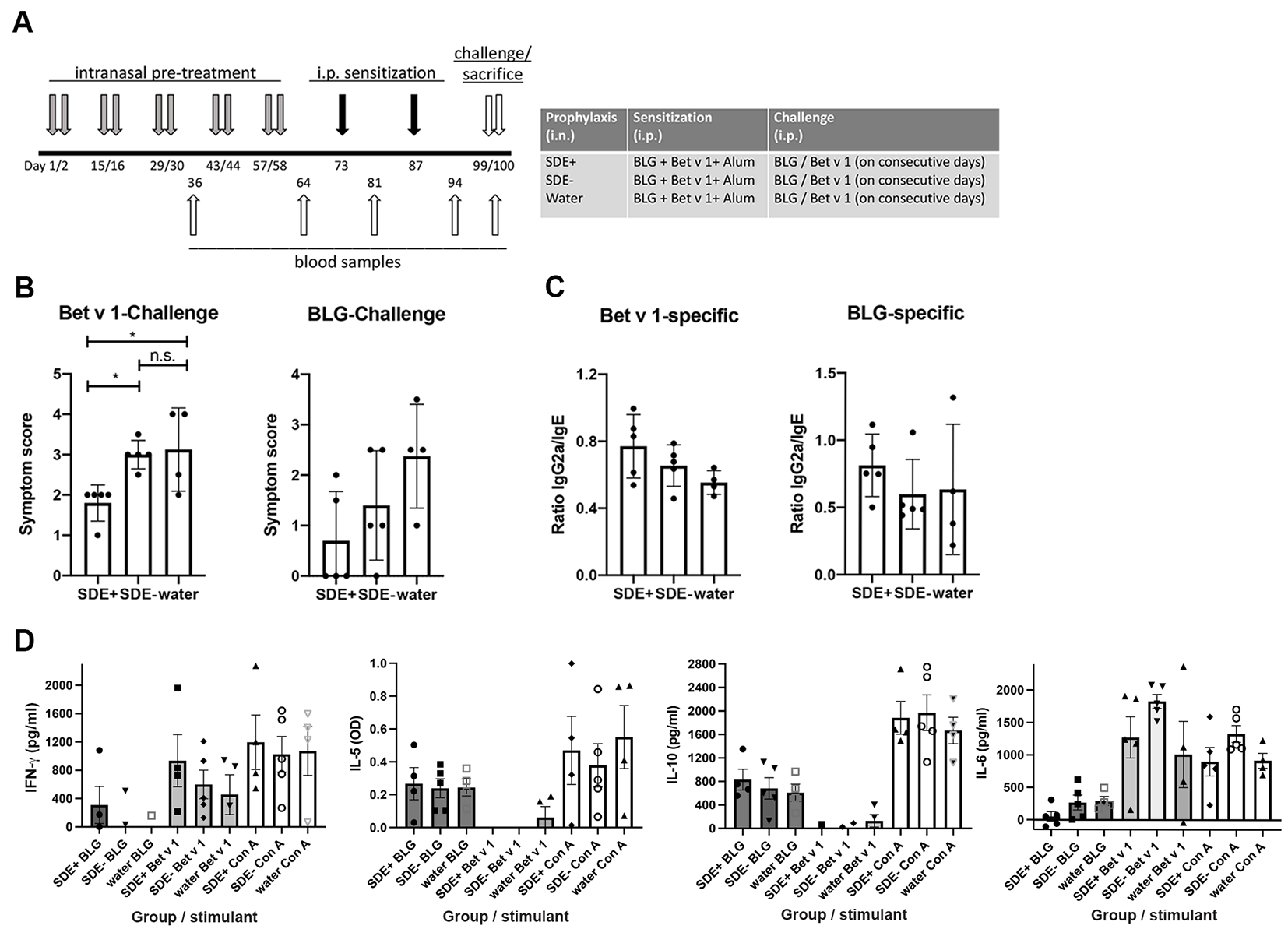

Supplement: Supplementary file 4 — Figure S3 [file CLT2-12-e12125-s005.tif]

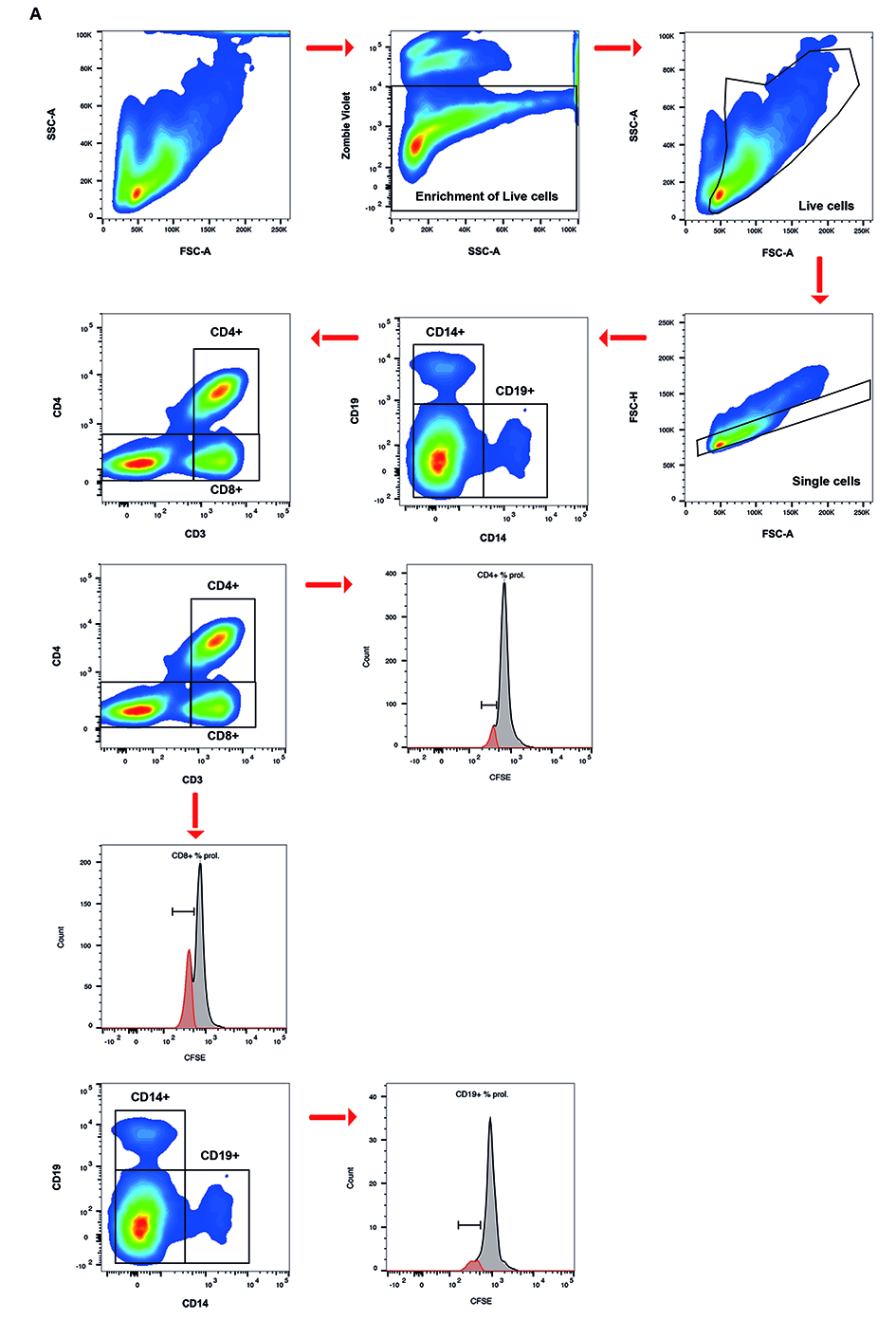

Supplement: Supplementary file 5 — Figure S4 [file CLT2-12-e12125-s003.tif]
